# Miniature-inverted-repeat transposable elements contribute to phenotypic variation regulation of rice induced by space environment

**DOI:** 10.3389/fpls.2024.1446383

**Published:** 2025-01-08

**Authors:** Lishan Chen, Qing Yang, Yan Zhang, Yeqing Sun

**Affiliations:** Institute of Environmental Systems Biology, College of Environmental Science and Engineering, Dalian Maritime University, Dalian, China

**Keywords:** rice, phenotype variation, TEs, mites, space environment, space mutagenesis, environment stress

## Abstract

**Introduction:**

Rice samples exposed to the space environment have generated diverse phenotypic variations. Miniature-inverted-repeat transposable elements (MITEs), often found adjacent to genes, play a significant role in regulating the plant genome. Herein, the contribution of MITEs in regulating space-mutagenic phenotypes was explored.

**Methods:**

The space-mutagenic phenotype changes in the F3 to F5 generations of three space-mutagenic lines from the rice varieties Dongnong423 (DN423) and Dongnong (DN416) were meticulously traced. Rice leaves samples at the heading stage from three space-mutagenic lines were subjected to high coverage whole-genome bisulfite sequencing and whole-genome sequencing. These analyses were conducted to investigate the effects of MITEs related epigenetic and genetic variations on space-mutagenic phenotypes.

**Results and discussion:**

Studies have indicated that MITEs within gene regulatory regions might contribute to the formation and differentiation of space-mutagenic phenotypes. The space environment has been shown to induce the transposable elements insertion polymorphisms of MITEs (MITEs-TIPs), with a notable preference for insertion near genes involved in stress response and phenotype regulation. The space-induced MITEs-TIPs contributed to the formation of space-mutagenic phenotype by modulating the expression of gene near the insertion site. This study underscored the pivotal role of MITEs in modulating plant phenotypic variation induced by the space environment, as well as the transgenerational stability of these phenotypic variants.

## 1 Introduction

The space environment, distinguished by its inherent complexity and pronounced mutagenicity, imparts profound multilevel impacts on organisms, frequently culminating in a spectrum of phenotype variations in model plants (Yu et al., 2007; Paul et al., 2012; Shi et al., 2014; Xu et al., 2021; Manzano et al., 2022). Elucidating the molecular mechanisms underlying mutagenesis induced by the space environment remains a central domain in the field of space biology. As manned spaceflight progresses, there is growing interest in using the space environment to enhance crop improvement and breeding programs (Xiao et al., 2010; Pei et al., 2019; Ma et al., 2021; Chen et al., 2024). However, the specific mechanisms underlying the formation of space-mutagenic phenotypes in plants and the stability regulation of these variations remain elusive, presenting a knowledge gap in our understanding of space biology.

Transposable elements (TEs), first discovered in maize in the 1940s by Barbara McClintock, were once labeled negatively as “junk DNA”, “selfish DNA” and “genomic parasites”. Researchers have gradually discovered that TEs possess a wide range of positive effects in the genome with the development of genetics and epigenetics (McClintock, 1984; Fedoroff, 2012; Casacuberta and González, 2013). TEs are the major component of the genome of most eukaryotes. Normally, epigenetic information targets TEs primarily and silences them. Epigenetic modification is susceptible to environmental changes, which may make TEs transcribe or even transpose actively, resulting in both epigenetic and genetic variation (Baduel and Quadrana, 2021; Klein and Anderson, 2022). TEs are characterized by their mobility and high proportion within eukaryotic genomes, facilitating recombination through homologous sequence recognition. Undoubtedly, these features make TEs an abundant source of genomic variation (Catlin and Josephs, 2022; Chuong et al., 2017; Schrader and Schmitz, 2019). In particular, TEs with high-copy numbers constitute the majority of the eukaryotic genomes and make a central contribution to the evolution of genes and genomes (Lisch, 2013).

Miniature-inverted-repeat transposable elements (MITEs) are a class of non-autonomous TEs that are incapable of encoding the enzymes necessary for their transposition. Typically, MITEs are shorter than 600 bp and are characterized by the presence of terminal inverted repeats (TIRs) at both ends. MITEs are predominantly found distributed along the chromosome arms, especially in the vicinity of genes, potentially influencing gene expression and regulatory functions (Chen et al., 2012; Fattash et al., 2013; Song and Cao, 2017). Accordingly, the impact of MITEs on gene expression regulation and plant phenotype diversity has garnered considerable interest (Domínguez et al., 2020; Castanera et al., 2021; Castanera et al., 2023). Recent genome-wide association study (GWAS) showed that TEs insertion polymorphisms (TIPs) of MITEs (MITEs-TIPs) could uncover more variations in crucial phenotypes during rice domestication than single nucleotide polymorphisms (SNPs) (Castanera et al., 2021). Additionally, GWAS revealed that TIPs, particularly MITEs-TIPs, were frequently associated with variations in gene expression during the evolution of rice, accounting for the majority of these associations. Many TIPs that regulate gene expression were positively selected during evolution (Castanera et al., 2023). Studies utilizing GWAS have exhibited great advantages in revealing the role of TEs in the generation of abundant genomic variation. However, these studies have provided a static “snapshot” of TEs based on genome sequencing data from various differentiated species, which limits our ability to observe in detail the dynamic regulation of TE activity across successive generations and its contribution to the stability of variation.

MITEs have been demonstrated to exert regulatory functions on a genome-wide scale through a variety of mechanisms. Changes in the epigenetic activity of MITEs, particularly those situated near coding genes and within regulatory regions, could significantly impact the regulation of adjacent genes and might also result in alterations of important agronomic traits in plants (Wei et al., 2014; Zhang et al., 2015; Shen et al., 2017; Xu et al., 2020; Gasparotto et al., 2023). Some MITEs contain their regulatory sequences, enabling them to function as enhancers or transcription factors (TFs) binding sites, thereby regulating the expression of adjacent genes (Hirsch and Springer, 2017; Morata et al., 2018). In addition to being located in the regulatory regions of genes and acting as controlling elements, MITEs could also be activated and undergo transposition under certain environmental conditions. This transposition might affect the regulation of genes near insertion sites in various ways. Transposition of MITEs could affect the expression of genes near the insertion site by introducing new regulatory sequences. Additionally, it could impact the expression of adjacent genes by altering DNA methylation patterns or influencing alternative splicing (Nakazaki et al., 2003; Naito et al., 2009; Morata et al., 2018; Dubin et al., 2018).

Previous space studies have demonstrated that the space environment can activate the transposition of the MITEs, named mPing, in the rice genome. And this variation can be inherited by subsequent generations (Long et al., 2009; Ou et al., 2009). Xu et al. discovered that the space environment induced hypomethylation of TEs in the Arabidopsis genome, a change that may correlate with the concurrent enhancement of TE activity attributable to space environment conditions (Xu et al., 2018, 2021). These studies demonstrated the effect of space environment on TEs activity. However, a notable gap exists in research exploring the influence of the space environment on phenotypic variation from the TEs perspective, underscoring the urgency for deeper exploration in this domain. Furthermore, little research has been reported that investigates the regulation role of TEs across successive generations and its impact on the phenotypic stability of plants.

Herein, rice materials from two rice varieties, Dongnong423 (DN423) and Dongnong416 (DN416), were selected as model plants. Notably, DN423 was identified as relatively more sensitive to the space environment than DN416, based on earlier research findings (Yu et al., 2007; Zeng et al., 2021). The rice samples were carried by SJ-10 retrievable science satellite, and the mutagenic rice plants were subsequently screened and identified upon return to Earth. The phenotypes of rice plant height, tiller number, and awn length are often related to their yield and environmental adaptability, and are therefore regarded as important phenotypes for study. Three space-mutagenic lines of rice, exhibiting variations in phenotypic traits mentioned above, were grown for three consecutive generations in the phytotron under standardized conditions. These space-mutagenic lines were cultivated simultaneously, with corresponding ground control samples included in each generation. Change patterns of space-mutagenic phenotype in F3 to F5 generations were meticulously tracked. Rice leaves at the heading stage were subjected to whole-genome sequencing (WGS) and whole-genome bisulfite sequencing (WGBS) with high coverage. Given the two potential mechanisms by which MITEs can affect genome stability, as discussed above, the extent of MITEs involvement in the process of space mutagenesis was explored in this study. It was shown that MITEs not only participated in the stability regulation of plant phenotypic diversity among offspring through epigenetic variation, but also contributed to phenotypic variation through insertion polymorphism triggered by space environment. Concurrently, the continuous multi-generation tracking of rice following its exposure to the space environment could offer insights into the adaptive evolutionary processes of plants under intense stress conditions. Therefore, this research not only contributed to an in-depth understanding of the mechanisms underlying phenotype diversity induced by the space environment but also shed further light on the crucial role of MITEs in regulating adaptive phenotype plasticity under stress conditions.

## 2 Materials and methods

### 2.1 Plant materials and experimental conditions

Rice cultivars of DN423 and DN416 were used in this study. The parental seeds of this study were carried aboard the SJ-10 retrievable satellite. The satellite orbited the Earth at an altitude of 252 kilometers with an inclination of 42 degrees for 12.5 days in 2016. The experimental materials for the parental generation of DN423 were derived from a single plant, while those for DN416 were obtained from another individual plant of the same variety. Each variety was divided into two equal portions, with one portion packaged in a bio-radiation box for launch via satellite, and the other portion stored in an identical box on the ground to serve as a control. The DN423 and DN416 seeds, retrieved from space, were planted at the Wuchang experimental base in Heilongjiang Province in 2016, alongside their corresponding ground control seeds. Space-mutagenic plants were identified in contemporary samples that had undergone space flight. The mutagenic plants, along with their corresponding ground control counterparts, were tracked and planted using the pedigree method. The specific procedure for the selection and breeding of space-mutagenic rice lines using the pedigree method was depicted in **Supplementary Figure 1**.

To ensure that multi-generational plant samples could be subjected to simultaneous biological analysis, specific space-mutagenic lines exhibiting distinct phenotypes of successive generations were selected. SA3-7, characterized by increased plant height and long awn, SA6-2, with an increased tiller number, and SC6-6, featuring a decreased tiller number, were identified following spaceflight. Seeds from the F2 to F4 generations of space-mutagenic lines were randomly collected and planted according to standard protocols in the phytotron, and plants from F3 to F5 generations were harvested.

Rice cultivation was standardized by the hydroponic method in the phytotron with a temperature of 28°C/25°C (day/night), the humidity of 70%, the light intensity of 300umol·m^-2^·s^-1^ and light duration of 14 h. The hydroponic nutrient solution was prepared using commercially available YOSHIDA rice nutrient solution (Coolaber) and pure water. Rice samples were planted for the full growth cycle. Rice leaves were collected at the heading stage, snap-frozen in liquid nitrogen and stored at -80°C until use. Samples from each treatment group were collected from three plants and divided into two parts, one for high-throughput sequencing and the other for RT-qPCR experiments.

### 2.2 Genomic DNA isolation

The CTAB method was used to extract gDNA from rice leaves. gDNA was quantified using Qubit Fluorimeter (Life Technologies). Nanodrop (Thermo Fisher Scientific) was used to verify DNA purity by measuring absorbance at 260/280 and 260/230 wavelengths. Agarose gel was used to verify DNA integrity. The same gDNA was used for whole-genome sequencing and whole-genome bisulfite sequencing for each sample.

### 2.3 Whole-genome sequencing

The library was constructed for each sample. The gDNA of all tissue samples was broken to an average length of 400 bp. The constructed library was subjected to paired-end sequencing of 2×150 bp reads on an Illumina Novaseq 6000 platform. And the sequencing depth was 40 ×. Fast QC (version 0.11.7) was used for quality control of raw sequencing reads, and adapter sequences and low-quality reads were removed. The alignment of high-quality reads to the reference genome (IRGSP-1.0) by using default parameters of BWA (version 0.7.12-r1039).

### 2.4 Whole-genome bisulfite sequencing

The gDNA was treated twice with bisulfite using EZ DNA Methylation-GoldTM Kit (Zymo Research) as manufacturer’s instructions. The library was constructed after purification. The constructed library was subjected to paired-end sequencing of 2×150 bp reads on an Illumina Novaseq 6000 platform. And the sequencing depth was 45 ×. Fast QC (version 0.11.7) was used for quality control of raw sequencing reads, and adapter sequences and low-quality reads were removed. The high-quality reads were aligned to the reference genome by Bismark (version 0.21.0), and bisulfite conversion efficiency was calculated. More than 99% of the cytosine in the rice genome was transformed in this study, indicating a very high bisulfite conversion rate. Methylated cytosines were extracted from the aligned reads with the default parameters of the Bismark.

### 2.5 Calculation of methylation level of MITEs

MITE-Tracker (Crescente et al., 2018) was used to extract and annotate MITEs from the rice genome. Methylation levels were calculated on all MITEs in each sample. The ratio of the number of methylated cytosines in an MITE to the total number of cytosines in the element was defined as the methylation level on the MITE. Differential methylation levels were calculated by comparing the methylation level of each space-mutagenic samples with the average methylation level of the corresponding three ground control lines (n = 3).

### 2.6 Detection of MITEs-TIPs

MITEs-TIPs detection was conducted on all 54 rice samples across the three rice space-mutagenic lines. High-coverage (40×) WGS data were aligned to the rice reference genome (IRGSP-1.0) using BWA (version 0.7.12-r1039). The soft-clipped generated during the alignment process were extracted. The positions where soft-clipped with a minimum alignment length of 10 bp, a minimum coverage depth of 5×, and target site duplication aligned were extracted as potential TIP loci. Further, these soft-clipped were aligned to a rice MITEs database extracted using MITE-Tracker. MITEs with soft-clipped alignments at both ends, indicating the presence of TIPs, were identified. The positions where these soft-clipped aligned within the reference genome were regarded as MITEs-TIPs loci and were used for further analysis. The schematic diagram of the detection method was shown in **Supplementary Figure 2**.

### 2.7 Gene ontology enrichment analysis

GO enrichment analysis was conducted at the GeneOntology.org. Oryza sativa was selected as the study species. The analysis was performed using the Fisher’s Exact Test. P-values were calculated to assess the enrichment of GO terms, and a threshold of P < 0.05 was applied to determine statistical significance. Gene lists used for GO enrichment analysis in this study were presented in **Supplementary Table 1**.

### 2.8 Genomic RNA isolation

Genomic RNA was extracted from rice leaves at the heading stage using FastPure Universal Plant Total RNA Isolation Kit (Vazyme) according to the manufacturer’s instructions. The concentration and purity of the extracted RNA were verified using Nanodrop. The RNA samples were stored at -80°C until use.

### 2.9 Quantitative real-time polymerase chain reaction

The RNAs were converted to cDNA using HiScript II Q RT SuperMix for qPCR with gDNA Eraser (Vazyme) according to the manufacturer’s instructions. qRT-PCR was performed on cDNA using ChamQ Universal SYBR qPCR Master Mix (Vazyme). The PCR reaction system was prepared according to the manufacturer’s instructions. The *OsActin1* (Os03g0718100) was used as reference gene, which was amplified by PCR under the same conditions. Each sample was repeated at least three times. The primer sequences used are shown in **Supplementary Table 2**. The ΔΔCt method was used to calculate the expression of the target gene in different samples.

### 2.10 Data visualization and statistical analyses

Bubble plots and bar charts in this study were drawn using ggplot2 (version 3.3.6) in R (version 4.1.2). GO chord plots were generated using the online bioinformatics platform SRplot (Tang et al., 2023). The chromosome distribution circle map of MITEs-TIPs was drawn by Circos (version 0.69-9). Pearson correlation was performed in R. Independent sample T-test and single sample T-test were performed in SPSS (version 23.0).

## 3 Results

### 3.1 Change patterns of rice space-mutagenic phenotypes in successive generations

**Figure 1:**
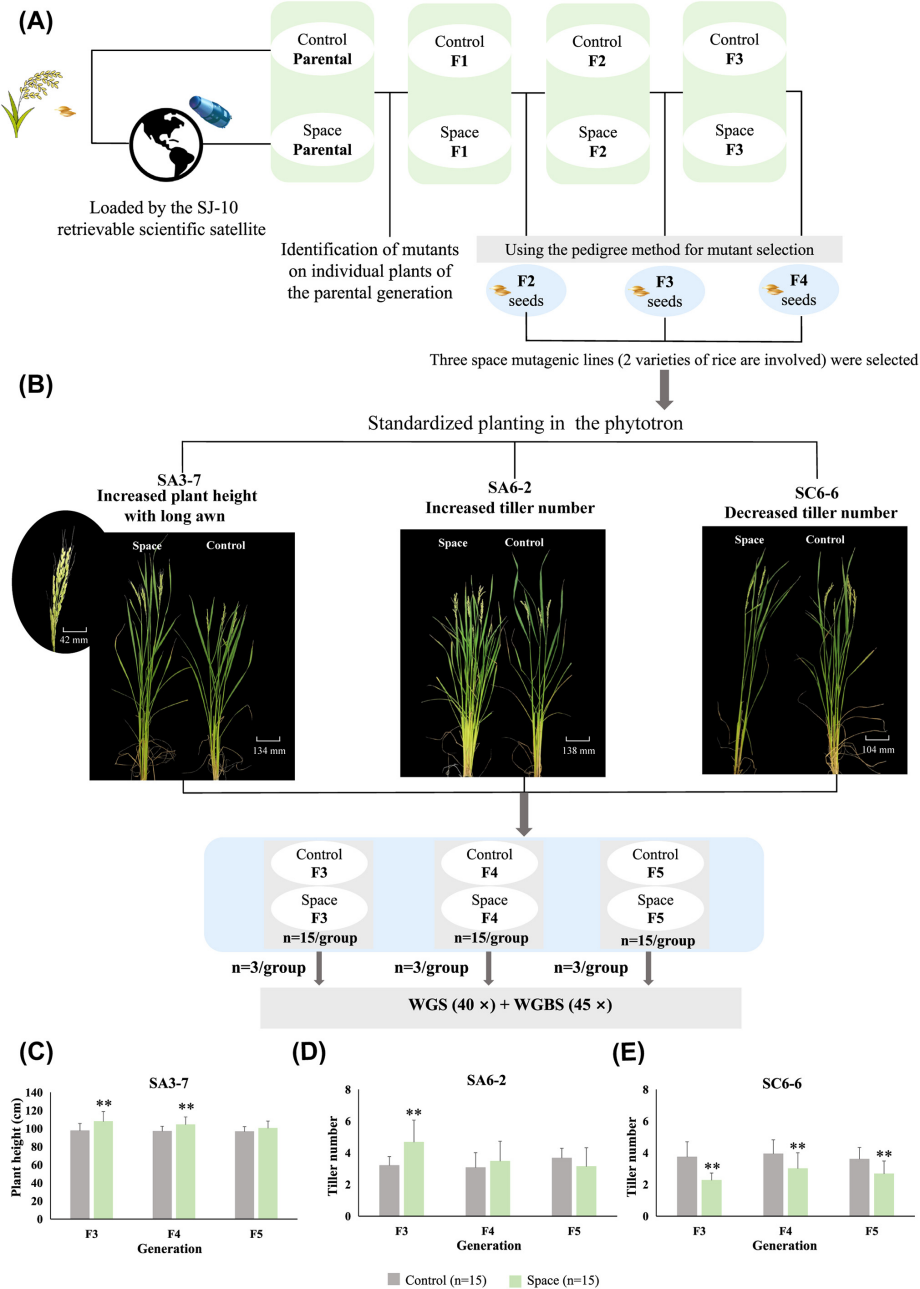
Acquisition of rice space-mutagenic lines and patterns of space-mutagenic phenotype change. **(A)** Schematic diagram of the acquisition and selection of space-mutagenic lines. The parental generation of space-mutagenic lines was carried by the SJ-10 retrievable scientific satellite. The space-mutagenic plants were identified within individual plants of the parental generation. The pedigree method was employed for mutant selection across subsequent generations. The mutant phenotypes of SA3-7 and SA6-2 were identified in the parental generation, while that of SC6-6 was identified in the F1 generation. Seeds from both space-mutagenic rice plants and corresponding ground control plants were harvested in F2-F4 generations; **(B)** Rice plants from different treatment groups in the F3-F5 generations of three space-mutagenic lines were cultivated in a standardized manner in the phytotron. Three plants were selected from each group for high coverage WGBS and WGS. The rice plants displayed here were all from the F3 generation; **(C)** The plant height in SA3-7 at F3-F5 generations; **(D)** The tiller number in SA6-2 at F3-F5 generations; **(E)** The tiller number in SC6-6 at F3-F5 generations. 15 strains per group were used as biological replicates; ** A highly statistically significant difference between the space and control samples using independent samples T-test (P < 0.01).

The space-mutagenic rice plants identified in individual plants from the parental materials carried by spacecraft were tracked and subsequently planted in the field for further study. Corresponding ground control plants were also included for comparison. The offspring of space-mutagenic plants were selected in the field using the pedigree method (**Figure 1A**). Three rice space-mutagenic lines, SA3-7, SA6-2, and SC6-6, were studied in this research, with a focus on the phenotype mutations involving plant height, awn length, and tiller number. In the parental plants that underwent space flight, two space-mutagenic rice lines were identified: SA3-7, characterized by increased plant height and long awn, and SA6-2, noted for its increased tiller number. Additionally, SC6-6, which presented with a decreased tiller number, was identified in the F1 generation. To observe the phenotype change patterns in the offspring of different space-mutagenic rice lines and to facilitate the simultaneous analysis of multiple generations, seeds from three successive generations (F2-F4) of space-mutagenic rice plants and their corresponding ground control samples were planted in the phytotron using the standard hydroponic method. For each group, 15 strains were utilized as biological replicates to ensure the reliability of the analysis (**Figure 1B**).

Distinct patterns of changes in space-mutagenic phenotype were observed in three rice space-mutagenic lines across successive generations from F3 to F5. The SA3-7 space-mutagenic rice line, characterized by an increase in plant height and the presence of long awn, consistently presented a taller plant height and longer awn phenotype compared to the ground control samples across successive generations up to F5. And no significant difference in plant height was observed between the space-mutagenic group and the control group in the F5 generation (**Figure 1C**). The increased tiller number phenotype of SA6-2 showed significant differences only in the F3 generation and recovered in the F5 generation (**Figure 1D**). The mutant phenotype of decreased tiller number isolated from F1 generation in SC6-6 has been maintained in offspring (**Figure 1E**).

### 3.2 Transgenerational change of methylation level in MITEs within gene regulatory regions of three rice space-mutagenic lines

**Figure 2:**
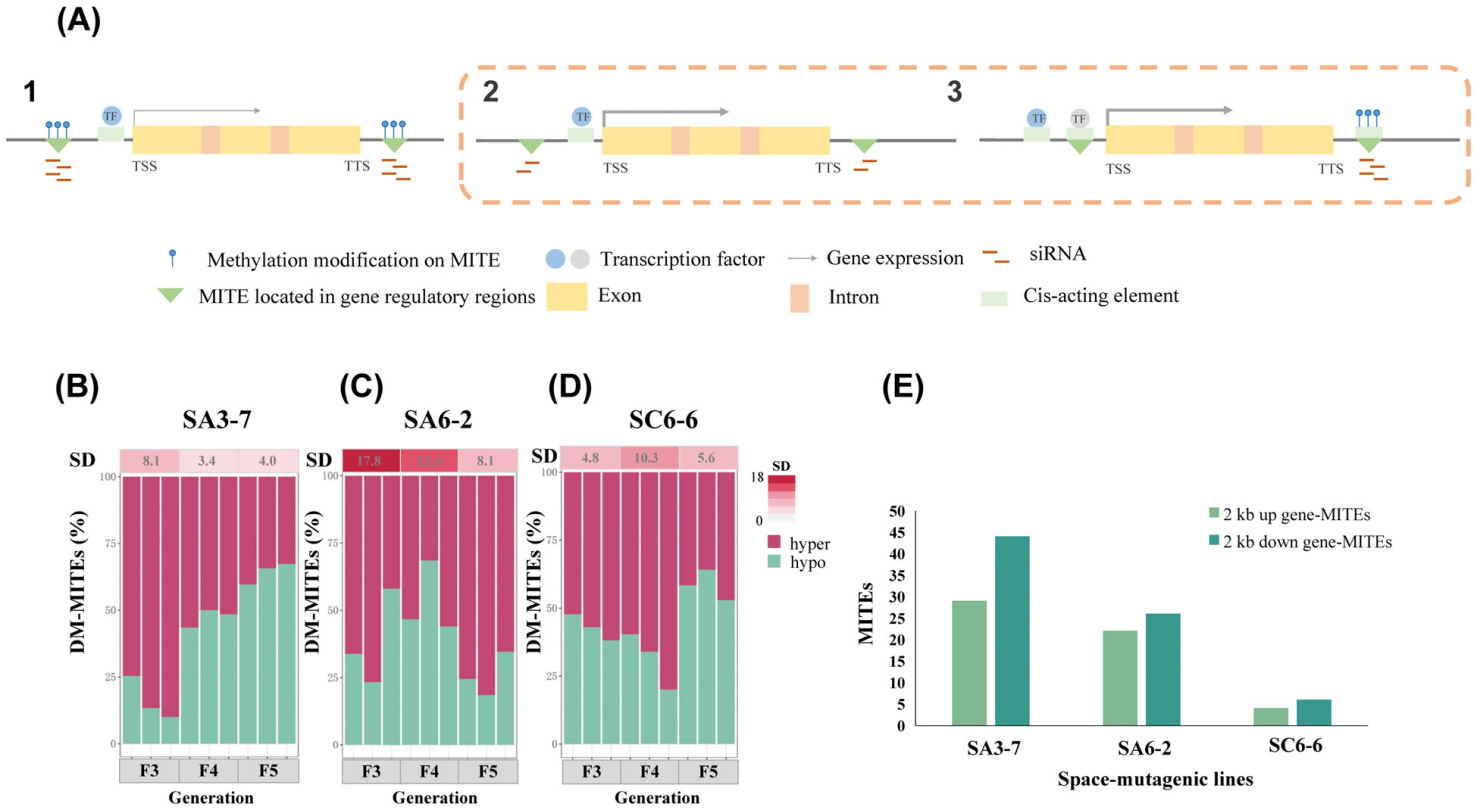
Analysis of MITEs methylation level changes within gene regulatory regions of three rice space-mutagenic lines. **(A)** 1. MITEs methylation modification and gene expression in gene regulatory regions under normal conditions. 2. Altered levels of siRNAs produced by MITEs in gene regulatory regions affect gene expression. 3. MITEs methylation modification in gene regulatory regions affects gene expression by affecting its binding to TFs. The thickness of the gray arrows represents the intensity of gene expression, the blue circle represents TFs that are bound to cis-acting elements, whereas the gray circle represents TFs that are bound to cis-acting elements located within MITEs; **(B)** The patterns of methylation level alterations on MITEs located within 2 kb upstream and downstream of genes in space-mutagenic rice samples across F3-F5 generations in SA3-7; **(C)** The patterns of methylation level alterations on MITEs located within 2 kb upstream and downstream of genes in space-mutagenic rice samples across F3-F5 generations in SA6-2; **(D)** The patterns of methylation level alterations on MITEs located within 2 kb upstream and downstream of genes in space-mutagenic rice samples across F3-F5 generations in SC6-6; Hyper and hypo represent the proportions of gene regulatory region MITEs in space-mutagenic rice samples with increased and decreased methylation levels, respectively. The heatmap above displays SD of differential methylation proportions in gene regulatory region MITEs among the three space-mutagenic samples per generation. Color intensity from light to dark represents increasing SD values. **(E)** The number of MITEs in gene regulatory regions that exhibit methylation level changes significantly and strongly correlated (P < 0.05, R > 0.65) with space-mutagenic phenotypes across generations in three rice space-mutagenic lines.

The methylation level alteration of MITEs within gene regulatory regions could affect the adjacent genes in a variety of ways as shown in **Figures 2A**. The transgenerational stability characteristics of methylation level alterations on MITEs within gene regulatory regions were analyzed in three rice space-mutagenic lines. A total of 9521 MITEs located 2 kb upstream and downstream of genes in the rice genome were extracted, including 4655 located upstream of genes and 4866 located downstream. The methylation levels of MITEs within the 2 kb upstream and downstream regions of genes were calculated for each sample. As shown in **Supplementary Table 3**, the methylation level differences of MITEs within gene regulatory regions were calculated for each space-mutagenic sample compared to its corresponding ground control samples. Subsequently, the proportions of MITEs within gene regulatory regions that exhibited increased methylation levels and those that showed decreased levels were analyzed for each space-mutagenic rice sample. The analysis aimed to uncover the patterns of methylation level alterations among MITEs within gene regulatory regions across all space-mutagenic rice samples (**Figures 2B–D**). To assess the stability of methylation level alterations on MITEs within gene regulatory regions, the standard deviation (SD) of the differential methylation proportion was calculated across different space-mutagenic rice samples. This analysis was performed for each generation across three space-mutagenic lines, emphasizing the consistency observed within individual generations. These results were depicted in the heatmap above **Figures 2B–D**. The results revealed that SA6-2 exhibited the highest SD value in the proportion of differential methylation of MITEs within gene regulatory regions in each generation (**Figure 2C**), with notable differences compared to SA3-7 (**Figure 2B**) and SC6-6 (**Figure 2D**). Moreover, a decreasing trend in the SD value was observed from F3 to F5 generations in SA6-2 (**Figure 2C**). Therefore, it was suggested that SA6-2 exhibited the most pronounced instability in methylation level alterations on MITEs within gene regulatory regions in each generation. Furthermore, the instability was observed to decline from F3 to F5 generation.

Further investigation was conducted to determine the correlation between methylation levels of MITEs within gene regulatory regions and the space-mutagenic phenotype across the F3 to F5 generations in three rice space-mutagenic lines. Pearson correlation analysis was conducted to evaluate the relationship between the methylation levels of MITEs within 2 kb upstream and downstream regions of genes and the corresponding space-mutagenic phenotypes in each rice space-mutagenic line. This assessment included individual samples from both space-mutagenic and ground control rice plants across F3 to F5 generations for each space-mutagenic line. Detailed site information for MITEs, whose methylation levels were found to be significantly and strongly (P < 0.05, R > 0.65) correlated with space-mutagenic phenotypes, as well as the corresponding gene site information, was presented in **Supplementary Table 4**. This included the methylation levels of these MITEs across each sample from the F3-F5 generations, along with the correlation coefficients and P values. The number of MITEs, located 2 kb upstream and downstream of genes, which were significantly and strongly correlated (P < 0.05, R > 0.65) with phenotypic changes across successive generations in each space-mutagenic line, was counted (**Figure 2E**). The results showed that the highest number of MITEs in gene regulatory regions, with methylation levels significantly and strongly correlated with transgenerational changes in space-mutagenic phenotypes, were identified in SA3-7, with SA6-2 and SC6-6 following in order (**Figure 2E**). These findings indicated that a greater number of MITEs within gene regulatory regions in SA3-7 might be implicated in the transgenerational regulation of space-mutagenic phenotypes through changes in methylation levels.

### 3.3 MITEs-TIPs variation in three rice space-mutagenic lines

**Figure 3:**
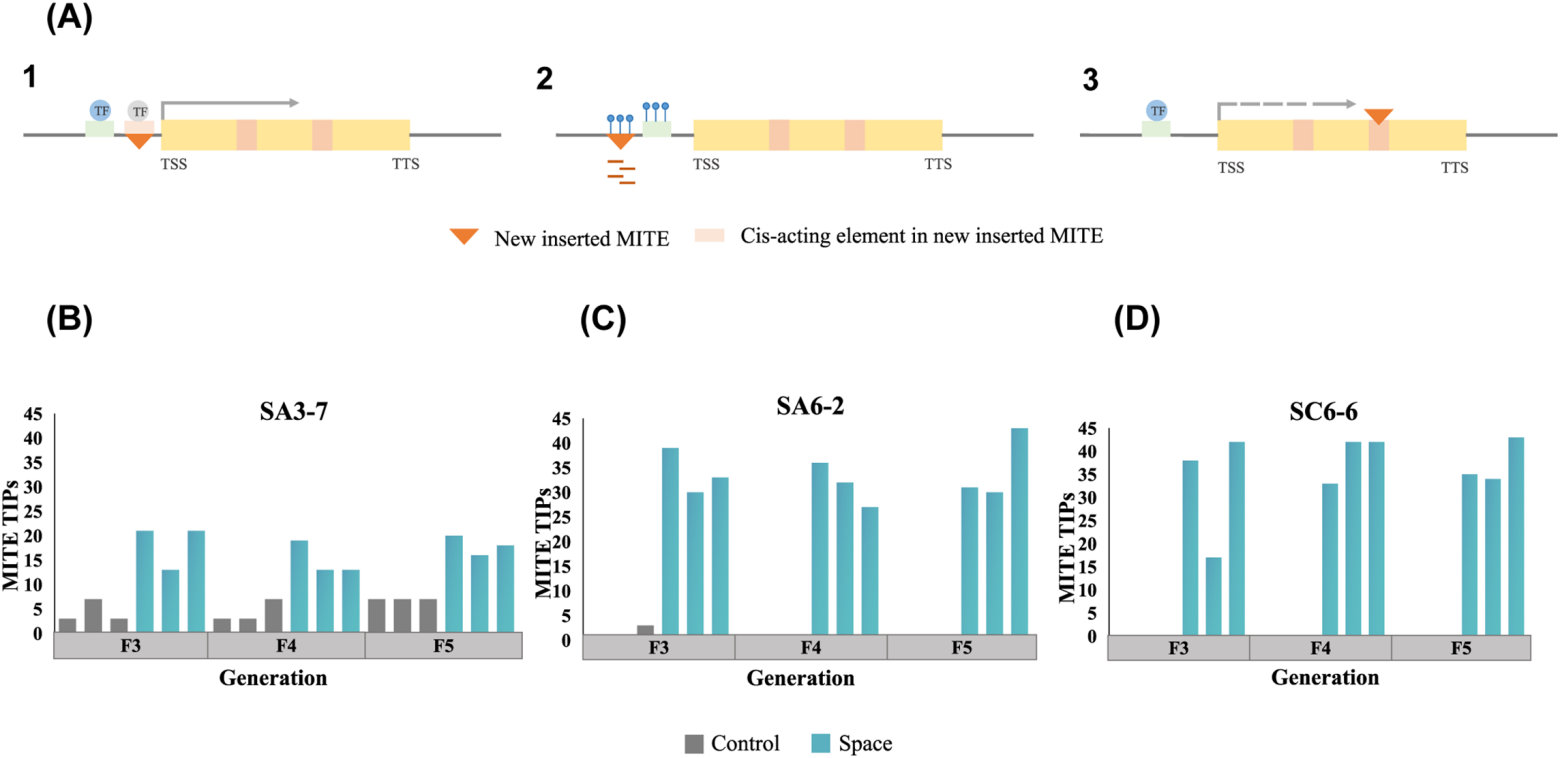
The detection of MITEs-TIPs variation in three rice space-mutagenic lines. **(A)** Several potential mechanisms in which MITEs transposition affects adjacent genes. Modules in the schematic diagram not specifically labeled represent the same meanings as those in **Figure 2A**. 1. A newly inserted MITE may provide cis-acting elements to an adjacent gene. 2. A newly inserted MITE may introduce a new epigenetic modification pattern to an adjacent gene. 3. Inserting a MITE into an intron can alter the expression of the adjacent gene; **(B)** A comparative analysis of MITEs-TIPs differences among different samples from F3 to F5 generations in SA3-7; **(C)** A comparative analysis of MITEs-TIPs differences among different samples from F3 to F5 generations in SA6-2; **(D)** A comparative analysis of MITEs-TIPs differences among different samples from F3 to F5 generations in SC6-6.

Previous research has shown that MITEs could not only serve as a “controlling element” at the original positions within gene regulatory regions, but also affect the genome via the creation of novel insertion sites (**Figures 3A**). To explore the contribution of MITEs to the genetic variation in three rice space-mutagenic lines, high throughput and high coverage WGS data were utilized to detect MITEs-TIPs. The specific methods and metrics for MITEs-TIPs detection were described in section 2.6. A random ground control sample from each generation served as a reference for initial comparisons to ensure a comprehensive capture of MITEs-TIPs variations across all rice samples. These comparisons assessed MITEs-TIPs differences among different rice samples within each generation. Subsequently, the presence of all detected differential MITEs-TIPs loci across three generations in each space-mutagenic line was reconfirmed within the genomes of the respective rice samples. The results showed that MITEs-TIPs were detected in all three rice space-mutagenic lines (**Figures 3B–D**), and detailed insertion site information was provided in **Supplementary Table 5**. MITEs-TIPs differences among ground control samples were also detected in SA3-7 and SA6-2 (**Figures 3B, C**), with a notably higher frequency of these differential sites observed in SA3-7 (**Figure 3B**). MITEs-TIPs were detected only in the space-mutagenic samples in SC6-6 (**Figure 3D**). The number of MITEs-TIPs differences detected in the space-mutagenic samples was higher than that detected in the ground control samples in all three space-mutagenic lines. It could be concluded that MITEs participated in the mutagenesis process of space environment by contributing genetic variation.

### 3.4 Distribution preference analysis of space-induced MITEs-TIPs

**Figure 4:**
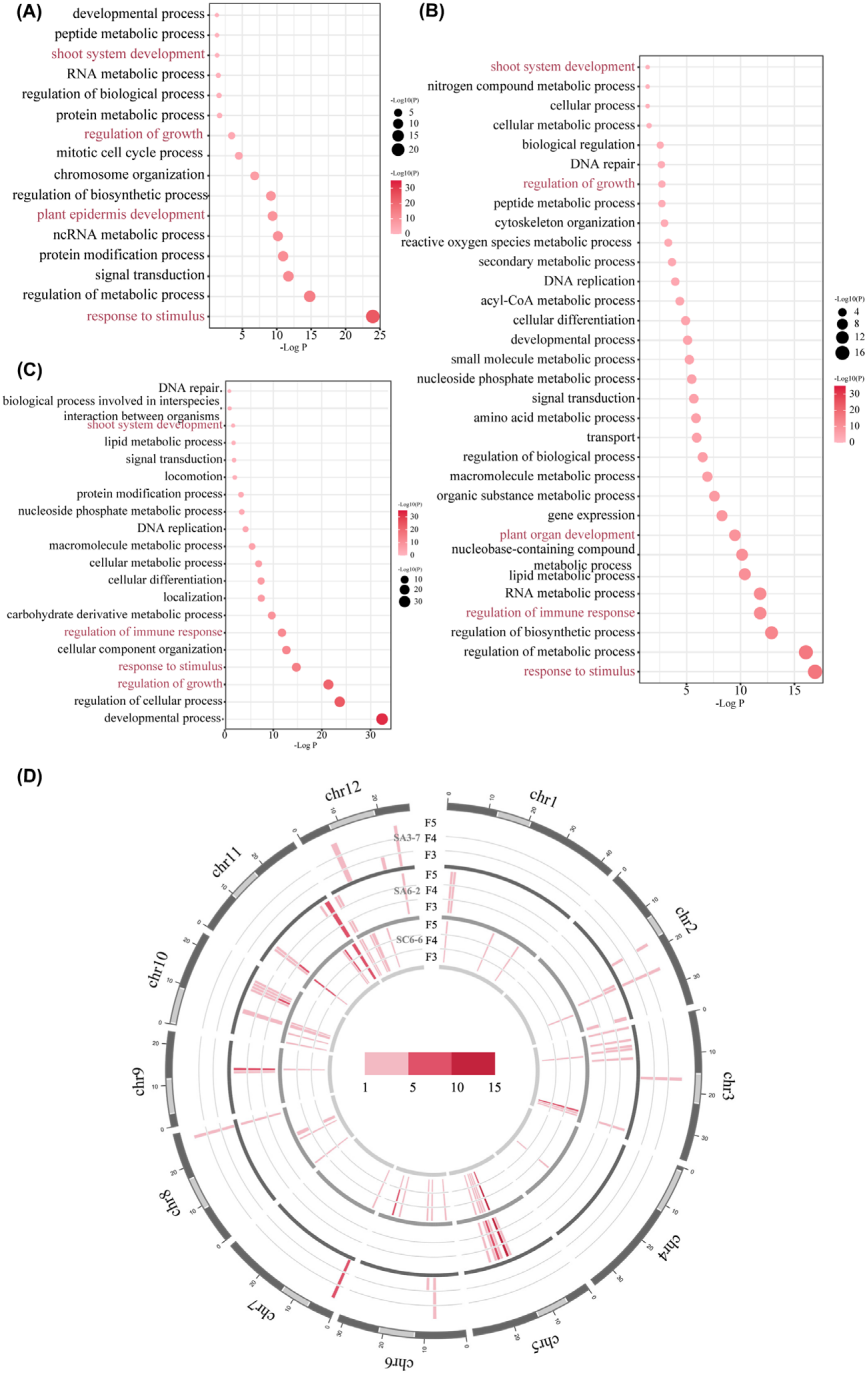
The distribution preference of space-induced MITEs-TIPs. **(A)** The bubble plot of GO enrichment analysis of genes near space-induced MITEs-TIPs in SA3-7; **(B)** The bubble plot of GO enrichment analysis of genes near space-induced MITEs-TIPs in SA6-2; **(C)** The bubble plot of GO enrichment analysis of genes near space-induced MITEs-TIPs in SC6-6; **(D)** The chromatin distribution of space-induced MITEs-TIPs in the three space-mutagenic lines; The outermost circle represents the 12 chromosomes of the rice genome, with light gray indicating heterochromatic regions and dark gray indicating euchromatic regions on each chromosome. From the inner circle to the outer circle, the chromatin distribution of space-induced MITEs-TIPs in SC6-6, SA6-2 and SA3-7 at F3-F5 generations were shown respectively. Each chromosome is divided into bins of 1,000,000 bp, with a color gradient from light pink to dark pink indicating the number of space-induced MITEs-TIPs detected in each bin.

MITEs-TIPs, uniquely detected in space-mutagenic samples after comparison with ground control samples, were induced by the space environment. The potential function of MITEs was closely related to the genomic background of the insertion sites. Therefore, adjacent genes were extracted for each space-induced MITEs-TIPs detected, including two genes located upstream and two genes located downstream relative to the MITEs-TIPs, along with the gene positioned at the site of the MITEs-TIPs. A gene set was formed from all genes adjacent to the space-induced MITEs-TIPs in each rice space-mutagenic line. These gene sets were then subjected to GO enrichment analysis to explore biological functions potentially affected by these MITEs-TIPs. The results revealed that space-induced MITEs-TIPs exhibited functional distribution preferences. Genes near the space-induced MITEs-TIPs were more likely to be significantly enriched in GO terms associated with phenotype changes and response to stimulus. For example, the “plant epidermis development” term was highly enriched in SA3-7 (**Figure 4A**). The “regulation of immune response” and “response to stimulus” terms were highly enriched in SA6-2 and SC6-6 (**Figures 4B, C**). These results indicated the potential contribution of MITEs in the generation of phenotypic diversity and stress response in plants after induced by space environment.

Given the unique functional distribution preferences of space-induced MITEs-TIPs, further analysis of the chromatin insertion preferences of these elements was conducted. The distribution frequency of space-induced MITEs-TIPs within euchromatin and heterochromatin regions was analyzed in three space-mutagenic lines (**Supplementary Table 6**). The delineation of euchromatic and heterochromatic regions was conducted in accordance with the findings reported by Cheng et al. (2001). The results indicated that the majority of space-induced MITEs-TIPs preferentially existed in the euchromatin region. In SA3-7, SA6-2, and SC6-6, 90.9%, 97.8%, and 89.8% of the space-induced MITEs-TIPs, respectively, were located in the euchromatic region. Furthermore, it was found that space-induced MITEs-TIPs showed insertion preferences on chromosome 7 (chr7) in SA3-7, and on chr5, chr9, and chr11 in SA6-2 and SC6-6 (**Figure 4D**). And this preference was determined by analyzing the number of space-induced MITEs-TIPs across different chromosomal bins in three rice space-mutagenic lines.

### 3.5 Effect of MITEs within gene regulatory regions on the stability of space-mutagenic phenotypes

To further elucidate the impact of methylation level alterations in MITEs within gene regulatory regions on the formation and differentiation of space-mutagenic phenotypes, a comprehensive analysis was conducted on the MITEs identified in section 3.2. These MITEs were observed to exhibit a significantly and strongly correlation (P < 0.05, R > 0.65) between their methylation levels and mutagenic phenotypic changes across generations. Genes corresponding to these MITEs were analyzed to identify key genes potentially affected by MITEs and involved in regulating the stability of space-mutagenic phenotypes.

**Figure 5:**
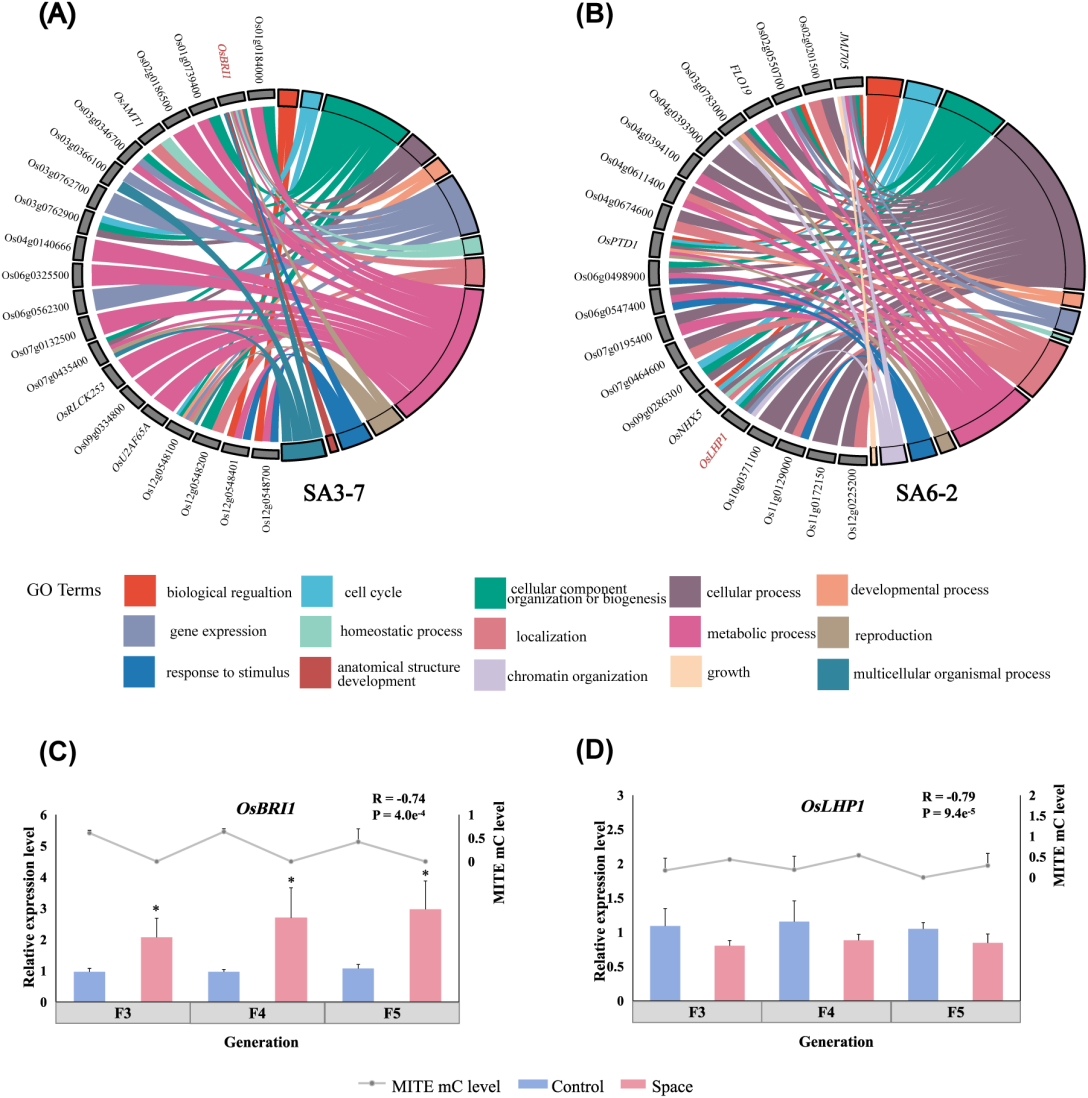
Identification and verification of the key genes involved in space-mutagenic phenotype changes under the influence of MITE methylation level change. **(A)** The chord plot of GO enrichment analysis for genes which the methylation level of regulatory regions MITEs significantly and strongly correlated (P < 0.05, R > 0.65) with change of plant height across generations in SA3-7; **(B)** The chord plot of GO enrichment analysis for genes which the methylation level of regulatory regions MITEs significantly and strongly correlated with change of tiller number across generations in SA6-2; Right side: significantly enriched GO terms (P < 0.05); Left side: corresponding genes. **(C)** *OsBRI1* expression levels and its downstream MITE methylation levels in SA3-7 across successive generations; **(D)** *OsLHP1* expression levels and its upstream MITE methylation levels in SA6-2 across successive generations; *A statistically significant difference between the space and control samples using independent samples T-test (P < 0.05).

GO enrichment analysis was performed to figure out the biological functions of these genes. The results showed that biological processes, including metabolic process, cellular component organization or biogenesis, and gene expression, were significantly enriched in SA3-7. Significantly enriched GO terms were associated with 21 genes, among which *OsBRI1* (Os01g0718300) was identified in the term of anatomical structure development, a process linked to phenotypic variation (**Figure 5A**). GO terms including cellular process, metabolic process and localization, were significantly enriched in SA6-2. Similar to SA3-7, the significantly enriched GO terms corresponded to a total of 21 genes in SA6-2 (**Figure 5B**). However, there were fewer significantly enriched GO terms and genes in SC6-6. As shown in **Supplementary Table 7**, these GO terms were mainly related to metabolic process, gene expression, and localization, and corresponded to 4 genes. Genes corresponding to significantly enriched GO terms were further queried. The functional query results of these genes were presented in **Supplementary Table 8**. Genes involved in regulating phenotypes associated with space-mutagenic phenotypes observed in this study were regarded as key genes. The results showed that *OsBRI1* (Os01g0718300), which was significantly enriched in anatomical structure development GO term of SA3-7, played a role in the regulation of rice plant height. Brassinosteroid (BR) were plant hormones that had been found to play a crucial role in the regulation of plant height in many grasses. Defects in BR synthesis or signaling usually resulted in plant dwarfing (Castorina and Consonni, 2020; Niu et al., 2022). There were three BR receptors in rice, *OsBRI1*, *OsBRL1* and *OsBRL3*. Among these, *OsBRI1* played a leading role in regulating plant height by influencing BR signal transduction and the elongation of rice internodes (Yamamuro et al., 2000; Nakamura et al., 2006; Zhao et al., 2013). *OsLHP1* (Os10g0324900), significantly enriched in chromatin organization and gene expression terms of SA6-2, was involved in the regulation of tiller number in rice. *OsLHP1* plays a pivotal role as a heterochronous gene. It modulated the plant’s developmental pace through epigenetic modification of key regulatory factors. Notably, the mutation of *OsLHP1* leaded to an increase in the number of tillers in rice (Cui et al., 2020). Further investigation was conducted to verify whether methylation changes of MITEs within gene regulatory regions of these genes play a role in the stability of space-mutagenic phenotypes.

Methylation level variations downstream of the *OsBRI1* gene across successive generations exhibited a strong and negative correlation with alterations in plant height within the SA3-7 (R = -0.68). The demethylation of MITE downstream of *OsBRI1* was observed across three successive generations of space-mutagenic samples as shown in **Supplementary Table 4**. The expression levels of *OsBRI1* were analyzed by qRT-PCR, revealing that the expression levels of *OsBRI1* were significantly higher in space-mutagenic samples compared to ground control samples in each generation. The expression levels of *OsBRI1* were significantly and negatively correlated with the change in downstream MITE methylation levels (**Figure 5C**). In addition, the methylation levels of MITE upstream of *OsLHP1* exhibited a strong positive correlation with tiller number changes in SA6-2 across generations (R = 0.76). Analysis revealed that *OsLHP1* expression was downregulated in space-mutagenic samples across three successive generations relative to ground control samples. The gene expression changes were significantly and negatively correlated with MITE methylation levels (**Figure 5D**). Therefore, it was suggested that changes in the methylation levels of MITEs within gene regulatory regions might contribute to the formation and stability regulation of space-induced mutagenic phenotypes, as supported by the observed correlations and gene expression patterns.

### 3.6 Expression analysis of genes involved in phenotypic regulation near space-induced MITEs-TIPs

In order to explore the relationship between space-induced MITEs-TIPs and the formation of space-mutagenic phenotypes, as well as to characterize their influence on nearby gene expression, we analyzed genes related to phenotypic regulation that were located near space-induced MITEs-TIPs in three rice space-mutagenic lines. Based on the functional distribution preference analysis of space-induced MITEs-TIPs in Section 3.4, genes corresponding to phenotype regulation-related GO terms were extracted. The detailed list of these genes, along with their potential roles and associations with space-induced MITEs-TIPs was presented in **Supplementary Table 9**.

**Figure 6:**
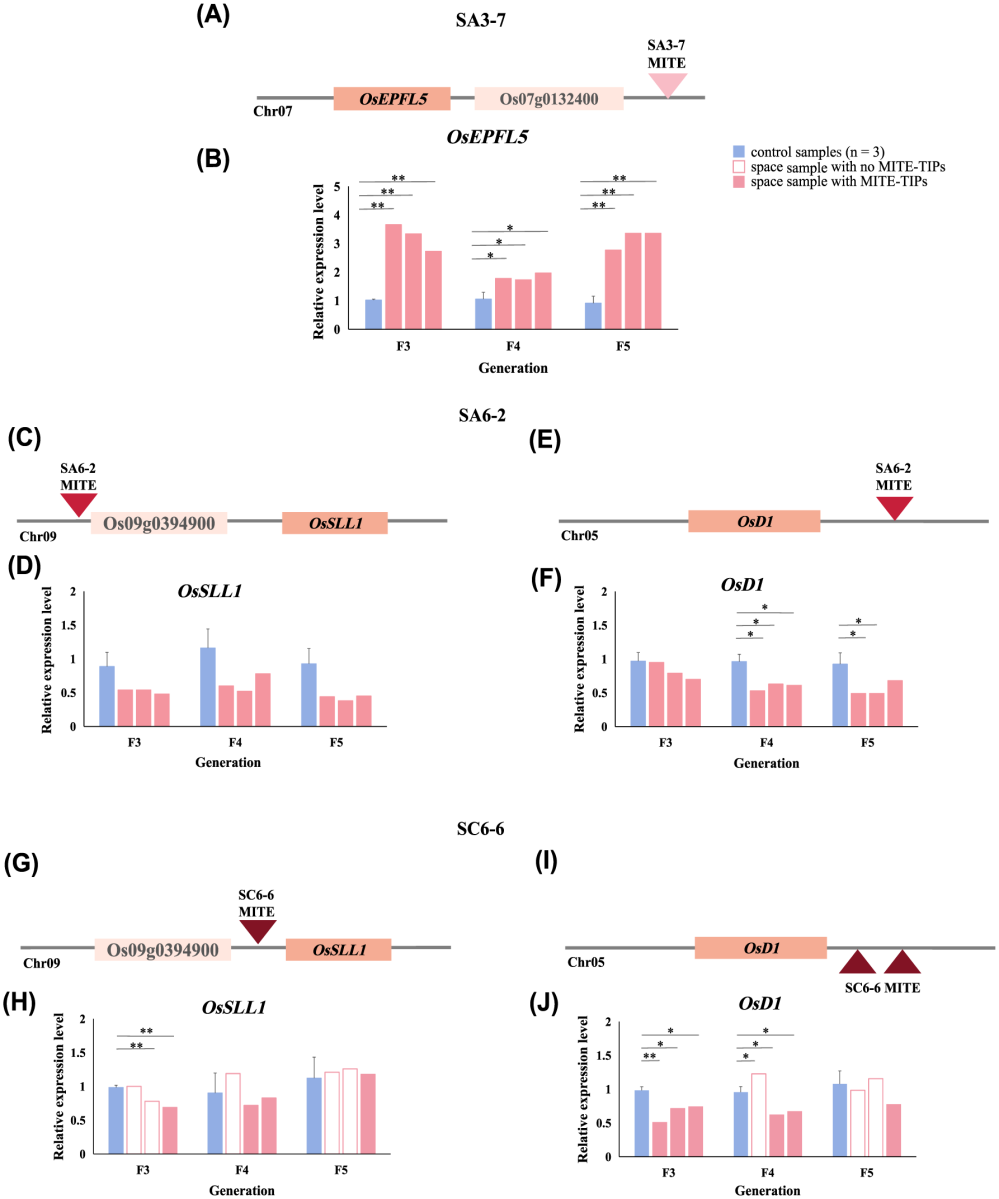
The expression levels of genes involved in phenotypic regulation near space-induced MITEs-TIPs. **(A)** Position relationship between *OsEPFL5* and space-induced MITEs-TIPs in SA3-7; **(B)** Relative expression levels of *OsEPFL5* in successive generations in SA3-7; **(C)** Position relationship between *OsSLL1* and space-induced MITEs-TIPs in SA6-2; **(D)** Relative expression levels of *OsSLL1* in successive generations in SA6-2; **(E)** Position relationship between *OsD1* and space-induced MITEs-TIPs in SA6-2; **(F)** Relative expression levels of *OsD1* in successive generations in SA6-2; **(G)** Position relationship between *OsSLL1* and space-induced MITEs-TIPs in SC6-6; **(H)** Relative expression levels of *OsSLL1* in successive generations in SC6-6; **(I)** Position relationship between *OsD1* and space-induced MITEs-TIPs in SC6-6; **(J)** Relative expression levels of *OsD1* in successive generations in SC6-6; *A statistically significant difference between the space and control samples using single sample T-test (P < 0.05), **A highly statistically significant difference between the space and control samples using single sample T test (P < 0.01).

The gene *OsEPFL5* identified in SA3-7, was demonstrated to be associated with the increase in rice plant height and the formation of the long awn phenotype. This finding aligned with the mutant phenotype characteristic of this rice space-mutagenic line. The space-induced MITEs-TIPs were identified 4752 bp downstream from the *OsEPFL5*. Notably, this insertion site was stably inherited across three successive generations of space-mutagenic samples (**Figures 6A, B**). Expression levels of *OsEPFL5* were analyzed to explore whether space-induced MITEs-TIPs could be implicated in the formation of space-mutagenic phenotype. The results demonstrated that the expression levels of *OsEPFL5* were significantly upregulated in space-mutagenic samples containing MITEs-TIPs (**Figure 6B**). Therefore, it was suggested that MITEs participated in the formation and maintenance of space-mutagenic phenotype by generating genetic variation.

*OsSLL1* detected in SA6-2 and SC6-6 near space-induced MITEs-TIPs was mainly associated with alteration in rice leaf morphology and the regulation of photosynthetic capacity, while *OsD1* mainly affected the regulation of plant height and drought tolerance in rice. Although space-induced MITEs-TIPs were detected near both *OsSLL1* and *OsD1* in these two space-mutagenic lines, their insertion sites and copy numbers were not identical. To investigate whether space-induced MITEs-TIPs could necessarily affect the expression of adjacent genes, the expression levels of *OsSLL1* and *OsD1* in the SA6-2 and SC6-6 space-mutagenic lines were analyzed. In SA6-2, MITEs-TIPs were located farther from the *OsSLL1* compared to their position in SC6-6. Despite this difference, space-induced MITEs-TIPs in SA6-2 were stably inherited across successive generations, unlike those upstream of *OsSLL1* in SC6-6, which exhibited instability in their inheritance (**Figures 6C, D, G, H**). *OsSLL1* expression levels of space-mutagenic samples harboring MITE insertion in SA6-2 were downregulated in each generation (**Figure 6D**). However, not all samples with detected MITEs-TIPs near *OsSLL1* in SC6-6 exhibited consistent differences in expression levels (**Figure 6H**). Space- induced MITEs-TIPs were detected downstream of *OsD1* in both SA6-2 and SC6-6, and two MITEs-TIPs were detected in SC6-6 (**Figures 6E, F, I, J**). Analysis of *OsD1* expression levels in SA6-2 and SC6-6 showed that not all samples displayed significant differences in expression, despite downregulation observed in samples with MITEs-TIPs (**Figures 6F, J**). These results suggested that while MITEs were prone to generating genetic variants near genes involved in phenotypic regulation following induction by the space environment, their impact on the expression of adjacent genes was not consistently significant.

## 4 Discussion

The exploration of the space environment has become increasingly frequent with the continuous advancement of manned space technology. Researchers have extensively investigated the potential impacts of the space environment on plants from diverse perspectives, which provides theoretical basis for understanding the effects of space environment on living organism (Zeng et al., 2020, 2022). The space environment is highly mutagenic and is known to induce a variety of phenotypic variations in model plants upon exposure to space conditions (Yu et al., 2007; Paul et al., 2012; Xu et al., 2021). Elucidating the causes of the highly mutagenic nature of the space environment will have significant implications for the safe and effective human utilization of space. TEs, as major constituents of eukaryotic genomes, are notably sensitive to environmental changes, thus serving as a significant source of genetic and epigenetic variation within plants genomes under various stress conditions (Catlin and Josephs, 2022; Chuong et al., 2017; Schrader and Schmitz, 2019). In this study, we conducted a thorough investigation into the function of the most prevalent class of TEs in rice, specifically the MITEs, focusing on their role in the formation and differentiation of space-induced mutagenic phenotypes.

Three rice space-mutagenic lines exhibiting phenotypic variation in plant height, tiller number, and awn length were utilized in this study. These specific phenotypes were targeted due to their well-established association with rice yield and environmental adaptability. Plant height, which was often associated with stress tolerance capabilities such as resistance to lodging, was observed (Gui et al., 2018). Tiller number, an important agronomic trait directly related to the reproductive capacity of rice, was also noted. The phenotype of long awn, predominantly found in wild varieties, has been observed to have gradually shifted to short awn or no awn during the process of domestication. This reduction in awn length was widely regarded as a significant step in the domestication and evolutionary history of rice. Moreover, the long awn phenotype in wild rice was primarily associated with resistance to biotic stress, which was an important aspect of the plant’s survival strategy (Xiong et al., 2022). Three successive generations of rice space-mutagenic lines were simultaneously cultivated in the phytotron using the hydroponics method. This controlled environment minimized the impact of varying planting conditions, ensuring that phenotypic data from different generations of rice samples could be reliably analyzed in parallel. In the analysis of transgenerational stability for three rice space-mutagenic lines from F3 to F5 generations, the space-mutagenic phenotypes of SA3-7 and SC6-6 were observed to be stable (**Figures 1C, E**), while SA6-2 showed a recovery pattern (**Figure 1D**). In comparison to traditional ground-based mutagenesis breeding methods, space mutagenesis breeding offered a broader range of variations and could achieve rapid stability, with some cases demonstrating stable traits by the F4 generation (Ma et al., 2021). This characteristic led to the space environment being regarded as an ideal source of mutagenesis for crop breeding research. However, the mechanisms behind the space environment’s high mutagenicity to plants, as well as the regulatory factors that govern mutagenic stability, have not been fully understood. The rice materials used in this study were of considerable value for further exploration of this topic.

The methylation level alteration patterns of MITEs within gene regulatory regions of each space-mutagenic rice sample were assessed. The stability of methylation level alterations on MITEs within gene regulatory regions of each generation was quantified by calculating the SD of the differential methylation proportion on these specific MITEs in the three space-mutagenic samples per generation (**Figures 2B–D**). As shown in **Figures 1C-E**, SA6-2 demonstrated the most pronounced transgenerational instability in mutant phenotypes compared to the other two rice space-mutagenic lines studied. This pattern was paralleled by the methylation level alterations of MITEs within the regulatory regions of SA6-2, which showed the greatest degree of instability across each generation. Moreover, this instability was observed to decrease with transgenerational inheritance (**Figure 2C**). MITEs, located within the gene regulatory regions of rice, exerted a broad influence on gene expression, playing a crucial role in plant growth and development (Lu et al., 2012). These elements could modulate the expression of neighboring genes through two primary mechanisms, the generation of siRNAs and by providing binding sites for TFs (Wei et al., 2014; Zhang et al., 2015; Xu et al., 2020; Gasparotto et al., 2023; Hirsch and Springer, 2017; Morata et al., 2018). Alterations in methylation levels on these MITEs often influenced their regulatory functions within the genome. Therefore, the observed consistency between the transgenerational stability of space-mutagenic phenotypes and the stability of methylation level alterations at MITEs within regulatory regions suggested that MITEs within gene regulatory regions might play a role in modulating the stability of space-mutagenic phenotypes.

The potential role of MITEs within gene regulatory regions in the formation or differentiation of space-mutagenic phenotypes was further investigated. Gene regulatory regions MITEs in which methylation levels were strongly correlated with phenotype changes across generations, and the genes corresponding to them were further analyzed. Two important genes, *OsBRI1* and *OsLHP1*, were identified in SA3-7 and SA6-2 respectively (**Figures 5A, B**). The correlation between the expression levels of these key genes and the methylation levels of MITEs in their regulatory regions was studied to determine the contribution of MITEs within gene regulatory regions to the formation and stability regulation of space-mutagenic phenotypes. In the SA3-7, which exhibited a space-mutagenic phenotype characterized by increased plant height and long awn, a stable inheritance pattern was observed (**Figure 1C**). Specifically, the demethylation of MITEs within 2 kb downstream of the *OsBRI1* gene was consistently maintained across three successive generations (**Supplementary Table 4**). Additionally, the expression levels of *OsBRI1* in the three successive generations of SA3-7 space-mutagenic samples were significantly higher than that of the corresponding ground control (**Figure 5C**). The variations in the expression levels of *OsBRI1* across successive generations were strongly negatively correlated with the alteration in methylation levels among MITEs located near *OsBRI1* (**Figure 5C**). Therefore, MITEs within gene regulatory regions might have participated in the formation and maintenance of plant height variation in SA3-7. SA6-2 was identified as the space-mutagenic line with increased tiller number in this study, and a recovery pattern was observed when the mutagenic phenotype of SA6-2 was tracked across successive generations in the offspring (**Figure 1D**). The change of methylation levels on MITEs within 2 kb upstream of *OsLHP1* across successive generations in SA6-2 was strongly correlated with the change in tiller number of samples (**Supplementary Table 4**). The expression levels of *OsLHP1* were downregulated in each generation of the space-mutagenic samples. The pattern of *OsLHP1* expression was strongly negatively correlated with changes in methylation levels of MITEs located near the *OsLHP1* across successive generations (**Figure 5D**). This result suggested that the methylation level change of MITEs located upstream of the *OsLHP1* might have played a role in the differentiation of space-mutagenic phenotype in successive generations of SA6-2. Therefore, alterations in the methylation levels of MITEs within gene regulatory regions were suggested to potentially contribute to the differentiation of space-mutagenic phenotypes across successive generations by influencing the expression of neighboring genes. Genes associated with the modulation of space-mutagenic phenotypes were identified using correlation analysis in this study. However, the number of key genes obtained was limited. For future research, developing computational software specifically for analyzing differential methylation of TEs, in combination with transcriptome sequencing, would be meaningful for further investigating the alterations in methylation levels of MITEs and their effects on the expression of neighboring genes and plant phenotypic variation.

The comprehensive examination of MITEs-TIPs in the three rice space-mutagenic lines were conducted in this research (**Figures 3B–D**). MITEs-TIPs that were uniquely identified in the genomes of space-mutagenic rice plants were extracted and have been classified as space-induced MITEs-TIPs (**Supplementary Table 6**). The presence of these space-induced MITEs-TIPs provided direct evidence for the involvement of MITEs in the genetic variation induced by the unique conditions of the space environment, suggesting a significant role for MITEs in space mutagenesis. Functional enrichment analysis of genes neighboring space-induced MITEs-TIPs revealed a distinct functional preference. The space-induced MITEs-TIPs preferred to distribute near genes associated with stress responses and those involved in the regulation of phenotypic traits (**Figures 4A–C**). Recent genomic studies on rice and tomato have also disclosed that TIPs, particularly MITEs-TIPs, showed a distribution preference near genes involved in environmental response, and these TIPs substantially influenced the modulation of agronomic traits (Domínguez et al., 2020; Castanera et al., 2021). Considering the important impact of MITEs-TIPs on plant phenotypic diversity, some researchers proposed that MITEs-TIPs could serve as valuable genetic markers for the improvement of crop breeding (Venkatesh and Nandini, 2020). Analysis of chromatin distribution preference showed that the vast majority of space-induced MITEs-TIPs were located in the euchromatin regions across the three space-mutagenic lines (**Figure 4D**). This euchromatin distribution preference of space-induced MITEs-TIPs was consistent with the distribution preference of MITEs themselves in the rice genome (Jiang and Panaud, 2013). And the propensity of MITEs to reside in euchromatin is often cited as a reason for their increased likelihood of influencing gene function compared to other types of TEs (Bennetzen and Wang, 2014; Schrader and Schmitz, 2019). MITEs insertion hotspots were identified on specific chromosomes, indicating a non-random distribution of these elements within the genome of rice space mutagenized samples (**Figure 4D**). As shown in **Supplementary Table 9**, we found that the genes involved in phenotypic regulation near the space-induced MITEs-TIPs were all situated within these hotspots. The findings from our analysis of the functional and chromatin distribution preferences of space-induced MITEs-TIPs have provided novel insights into the potential regulatory roles of MITEs. Specifically, they contributed to the modulation of plant stress responses and the diversification of phenotypes after exposed to space conditions.

SA3-7 was a rice space-mutagenic line characterized by increased plant height and long awn phenotype variation studied in our research. Its space-mutagenic phenotype was stably maintained across the observed F3 to F5 generations (**Figures 1B, C**). Based on the functional preference analysis, we identified that a space-induced MITEs-TIPs in the SA3-7 was located downstream of the *OsEPFL5* gene, which might be implicated in the regulation of rice awn length and plant height (**Supplementary Table 9**). Specifically, this MITEs-TIP was identified within a prominent insertion hotspot on chr07. The stable inheritance of MITEs-TIPs downstream of the *OsEPFL5* gene in SA3-7 space-mutagenic samples corresponded to the stable inheritance of the increased plant height and long awn mutagenic phenotype across three successive generations. Plant intercellular communication helps coordinate development and environmental response. Small secretory peptides (SSPs) signals mediate intercellular signal transduction during the development and pattern formation of multicellular organisms. SSPs encoded by EPF/EPFL family genes, known as Epidermal patterning factors and their like proteins, have proven to regulate awn development during rice domestication (Xiong et al., 2022). And another study also indicated that *OsEPFL5* might positively regulate rice plant height (Guo et al., 2023). We conducted an analysis of the *OsEPFL5* gene expression levels across three successive generations of the SA3-7 space-mutagenic line. The results revealed a significant upregulation of the *OsEPFL5* gene expression in the space-mutagenic samples of each generation (**Figure 6B**). This implied that space-induced MITEs-TIPs might modulate the expression of *OsEPFL5*, thereby contributing to the formation of the space-mutagenic phenotype of increased plant height and awn length in SA3-7. It could be concluded that the space-induced MITEs-TIPs is one of the important causes for the high mutagenicity of space environment.

Space-induced MITEs-TIPs have been identified in proximity to genes involved in phenotypic regulation in the SA6-2 and SC6-6. Genes such as *OsSLL1* and *OsD1* have been found to be located near these MITEs-TIPs (**Supplementary Table 9**). However, the previously reported phenotypic regulatory functions of these genes did not show a direct association with the phenotypic variation of tiller number observed in this study. For example, *OsSLL1* was mainly involved in modulating rice leaf morphology and regulating photosynthetic capacity (Zhang et al., 2009; Ren et al., 2019), and *OsD1* mainly associated with the regulation of rice plant height and the plant’s drought tolerance (Ferrero-Serrano and Assmann, 2016; Ferrero-Serrano et al., 2018; Li et al., 2023). The expression levels of these two genes in SA6-2 and SC6-6 were analyzed to explore the impact of space-induced MITEs-TIPs on gene expression (**Figures 6D, F, H, G**). The results showed that space-induced MITEs-TIPs did not necessarily have a significant impact on the expression of nearby genes. Moreover, the proximity of MITEs-TIPs to genes did not correlate with a higher likelihood of affecting gene expression. Regarding the fact that not all space-induced MITEs-TIPs significantly affect adjacent genes, we speculated that there might be several reasons, as described in the following points. Firstly, the effect of MITEs-TIPs from the perspective of gene expression level was analyzed in this study, and there were also studies showing that MITEs might affect genes at the level of translation (Shen et al., 2017). Secondly, some MITEs might have a neutral impact on genes, which means, insertion itself would not have a significant impact on gene function in order not to be eliminated by the genome and remain in the genome (Chuong et al., 2017; Bourque et al., 2018). Offspring of different space-mutagenic lines might undergo different selective effects in natural environment. Some of these effects might be fixed or retained in the genome, while others might be silenced by selection, resulting in their presence at low frequencies in the offspring’s genome (Castanera et al., 2021). However, it was undeniable that the space-induced MITEs-TIPs tended to be distributed in the euchromatin region and near the genes involved in phenotype regulation, which would bring the possibility of abundant variation to the genome.

Three rice space-mutagenic lines were examined in this study, which are categorized under two rice varieties, DN423 and DN416. Specifically, the SA3-7 is categorized under the DN423 variety, and SA6-2 and SC6-6 were classified under the DN416 variety. DN423, formerly known as DongnongV7, was identified as a space-sensitive rice variety in earlier studies because the changes in its space-mutagenic phenotypes were more readily detectable (Yu et al., 2007; Zeng et al., 2021). However, the underlying molecular mechanisms have not yet been explored. We hypothesized that MITEs might be related to the differences of space sensitivity exhibited in different rice cultivars, mainly for the following reasons. It was determined in our study that the effects of MITEs on genes related to the specific mutagenic phenotype were more readily identifiable in the SA3-7 than in the two other DN416 rice space-mutagenic lines (**Figures 5C**, **6B**). Additionally, a higher number of MITEs within gene regulatory regions, which exhibited a strong correlation between methylation level alterations and space-mutagenic phenotype changes across generations, were observed in SA3-7. The quantity of these MITEs surpasses that observed in both SA6-2 and SC6-6 (**Figure 2E**). This implied that a potentially higher abundance of MITEs in SA3-7 compared to SA6-2 and SC6-6 might have played a role in the formation or maintenance of the space-mutagenic phenotype. Furthermore, a greater number of MITEs-TIPs differences were also identified among the ground control rice samples in SA3-7, indicating relatively high variability of MITEs in DN423 genome (**Figure 3B**).

## 5 Conclusion

Phenotypic variations have been induced in the rice samples following spaceflight, and continuous tracking of their offspring has revealed diverse patterns of phenotype stability across successive generations. Genetic and epigenetic variations associated with MITEs in three rice space-mutagenic lines, which exhibited different phenotypic stability, were systematically analyzed in this study. Consistency was observed between the stability of methylation level alterations on MITEs and the phenotypic stability of rice space-mutagenic lines when examining the patterns of methylation changes on MITEs within gene regulatory regions across F3 to F5 generations. Further correlation analysis identified key genes responsible for regulating space-mutagenic phenotypes. Furthermore, variations in the expression levels of these genes across generations demonstrated a significant correlation with the methylation level alterations in their regulatory region MITEs. This finding confirmed that MITEs might serve as controlling elements within gene regulatory regions after exposure to space environmental stress, participating in the regulation of the stability of space-mutagenic phenotypes through dynamic changes in their methylation levels. MITEs contributed to the genetic variation in space-mutagenic rice samples by forming TIPs after induction by the space environment. These space-induced MITEs-TIPs tended to insert near genes involved in phenotypic regulation and stress response, and influenced the expression levels of key genes that regulate space-mutagenic phenotypes. The results collectively indicated that MITEs played a pivotal role in the process of space mutagenesis, highlighting their potential value for future research on space mutagenesis breeding.

Meanwhile, the regulation of plant phenotypes is a process that involves complex genetic networks. A limitation of our study lies in its inability to delve deeply into the intricate mechanisms through which MITEs contribute to these complex gene regulatory networks during space mutagenesis. To mitigate this limitation in future research, integrating WGS and WGBS data with transcriptome sequencing data will offer valuable insights, fostering a more comprehensive understanding of these processes. In summary, the current research has deepened the understanding of the mechanisms behind space mutagenesis and has highlighted the crucial role of MITEs in fostering plant phenotype diversity and plasticity in response to severe environmental stress.

## Data Availability

The datasets presented in this study can be found in online repositories. The names of the repository/repositories and accession number(s) can be found in the article/**Supplementary Material**.

## Supplementary material

Supplementary Figure 1 Breeding process of rice space-mutagenic lines using a pedigree method.

Supplementary Figure 2 Schematic diagram of MITEs-TIPs detection.

Supplementary Table 1 Gene lists used for GO enrichment analysis.

Supplementary Table 2 Primers used in this paper for qRT-PCR.

Supplementary Table 3 MITEs in gene regulatory regions with methylation level alterations.

Supplementary Table 4 Pearson correlation analysis of methylation levels on MITEs within gene regulatory regions and space-mutagenic phenotypes in three rice space-mutagenic lines.

Supplementary Table 5 MITEs-TIPs variation in three rice space-mutagenic lines.

Supplementary Table 6 Space-induced MITEs-TIPs in three rice space-mutagenic lines.

Supplementary Table 7 GO enrichment analysis of genes with strong and significant correlations between MITEs methylation levels in regulatory regions and space-mutagenic phenotypes.

Supplementary Table 8 Functional query of genes corresponding to important regulatory region MITEs in three rice space-mutagenic lines.

Supplementary Table 9 Genes associated with phenotypic regulation in the vicinity of space-induced MITEs-TIPs.
