# Supplementary material for: Miniature-inverted-repeat transposable elements contribute to phenotypic variation regulation of rice induced by space environment
Source: Front Plant Sci. 2025 Jan 8;15:1446383. doi: 10.3389/fpls.2024.1446383 (PMC11751223; doi:10.3389/fpls.2024.1446383)
Supplement: Supplementary Figure 1 — Breeding process of rice space-mutagenic lines using a pedigree method. [file DataSheet1.zip › Supplementary Material/Supplementary Figure 1.docx]

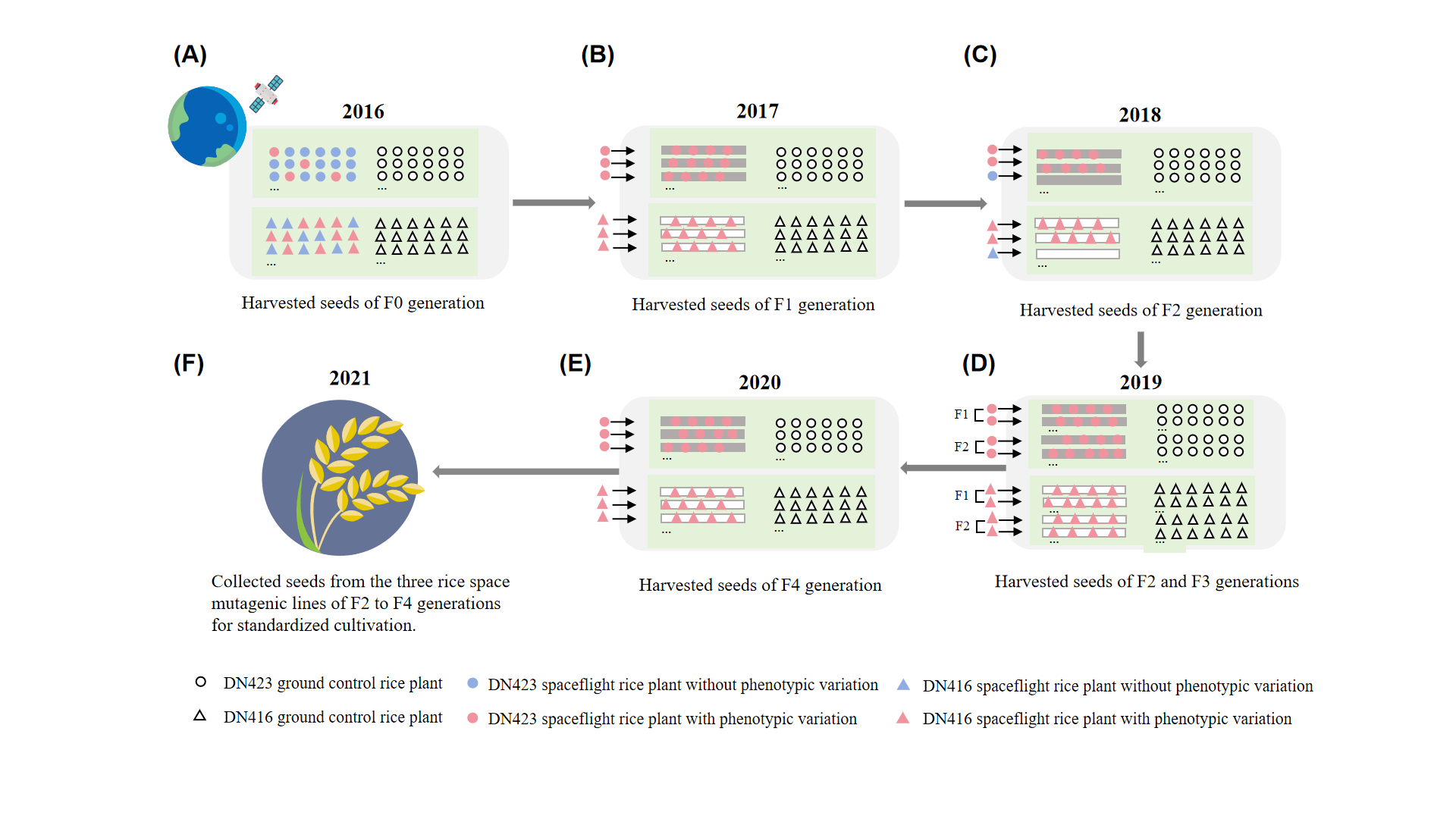


Supplementary Figure 1. Breeding process of rice space-mutagenic lines using a pedigree method. (A) In 2016, rice seeds that had undergone spaceflight and their corresponding ground control seeds were simultaneously cultivated in the field. And space-induced mutagenic rice plants were identified within individual plants; (B) In 2017, all seeds harvested from space-mutagenic rice plants were planted in row; (C) In 2018, all rice seeds harvested in 2017 were planted in row for tracking and evaluation; (D) In 2019, rice seeds harvested in 2017 and 2018 were planted simultaneously. For ease of identification, sampling, and seed conservation within a large sample population, each space-mutagenic rice line previously obtained was tracked by planting 30 rows; (E) In 2020, the F3 generation rice seeds harvested in 2019 were tracked by planting 30 rows for each lineage; (F) In 2021, seeds of three rice space-mutagenic lines from the F2 to F4 generations were selected for standardized cultivation in the phytotron. The ground control rice materials were planted simultaneously with the spaceflight rice plants and propagated through generations for each rice space mutagenic line.
