# Supplementary material for: Miniature-inverted-repeat transposable elements contribute to phenotypic variation regulation of rice induced by space environment
Source: Front Plant Sci. 2025 Jan 8;15:1446383. doi: 10.3389/fpls.2024.1446383 (PMC11751223; doi:10.3389/fpls.2024.1446383)
Supplement: Supplementary Figure 1 — Breeding process of rice space-mutagenic lines using a pedigree method. [file DataSheet1.zip › Supplementary Material/Supplementary Figure 2.docx]

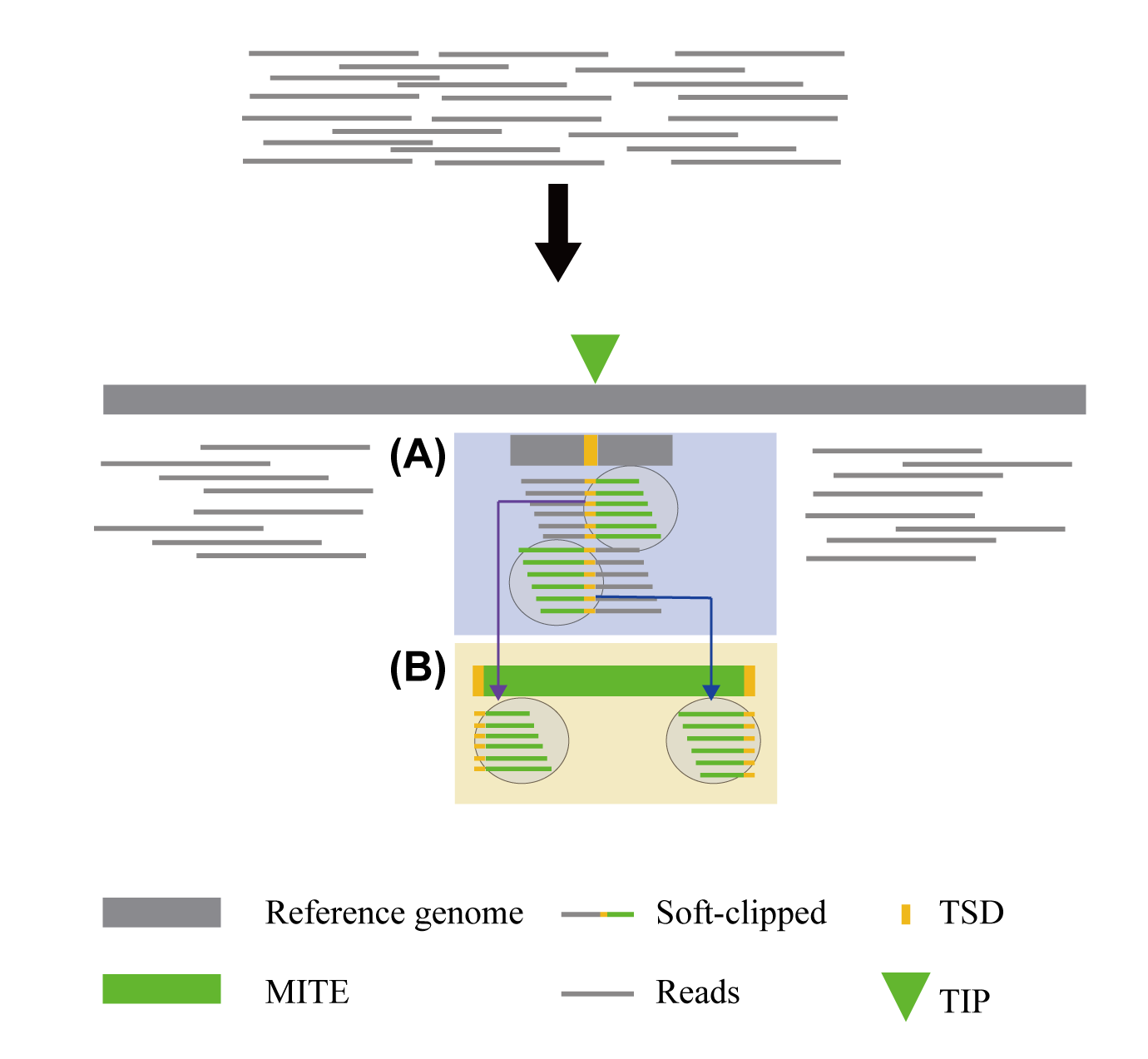


Supplementary Figure 2. Schematic diagram of MITEs-TIPs detection. (A) Extraction of potential TIPs loci. During the alignment of WGS data to the rice reference genome, the positions where soft-clipped with a minimum alignment length of 10 bp, a minimum coverage depth of 5×, and target site duplication aligned were extracted as potential TIP loci. (B) The soft-clipped sequences, corresponding to potential TIPs loci, were aligned to the rice MITEs database.
