# Supplementary material for: Miniature-inverted-repeat transposable elements contribute to phenotypic variation regulation of rice induced by space environment
Source: Front Plant Sci. 2025 Jan 8;15:1446383. doi: 10.3389/fpls.2024.1446383 (PMC11751223; doi:10.3389/fpls.2024.1446383)
Supplement: Supplementary Figure 1 — Breeding process of rice space-mutagenic lines using a pedigree method. [file DataSheet1.zip › Supplementary Material/Supplementary Table 8.docx]

Supplementary Table 8. Functional query of genes corresponding to important regulatory region MITEs in three rice space-mutagenic lines.

| **Gene ID^a^** | **Gene symbols** | **MITE^b^ Location** | **R** | **Verified / putative gene function** | **Reference** |
| --- | --- | --- | --- | --- | --- |
| **SA3-7** | | | | | |
| Os12g0548100 | - | Down | -0.73 | Encoding HEAT repeat family protein | - |
| Os01g0718300 | *OsBRI1*, *d61*, *SMG12* | Down | -0.68 | The BR signal receptor in rice regulates the organ development of rice. After mutation of this gene, the typical phenotypes of rice include restricted internode elongation, reduced plant height, decreased leaf angle, and upright leaf posture, etc. | Yamamuro et al., 2000、Nakamura et al., 2006、Zhao et al., 2013. Niu et al., 2022 |
| Os03g0346700 | - | Down | 0.66 | Encoding jmjC domain containing protein | - |
| Os12g0548700 | - | Up | -0.71 | Encoding inhibitor I family protein | - |
| Os12g0548401 | - | Down | -0.72 | Encoding inhibitor I family protein | - |
| Os03g0762900 | - | Down | 0.66 | - | - |
| Os01g0184000 | - | Down | 0.72 | - | - |
| Os07g0435400 | - | Down | 0.73 | Encoding G-beta repeat domain containing protein | - |
| Os12g0548200 | *XB21* | Up | -0.73 | Encoding auxilin-like protein. The overexpression of this gene will upregulate the expression of genes related to endocytosis, enhancing the resistance of rice to Xanthomonas oryzae pv. Oryzae. | Park et al., 2017 |
| Os01g0739400 | - | Up | 0.66 | - | - |
| Os03g0762700 | - | Up | 0.70 | - | - |
| Os06g0562300 | - | Up | -0.69 | Encoding homeobox domain containing protein | - |
| Os03g0366100 | - | Down | -0.65 | - | - |
| Os02g0620500 | *OsAMT1; 3*, *OsAMT1.3*, *OsAMT1; 2* | Up | -0.71 | The gene encodes an ammonium transporter, affecting the absorption and utilization of nitrogen by the rice root system. After the gene knockout, there is a reduction in the length of the seminal root, the total number of lateral roots, and the length of the lateral roots. | Sonoda et al., 2003、Konishi and Ma., 2021、Li et al., 2022 |
| Os02g0186500 | - | Down | 0.66 | Encoding BRASSINOSTEROID INSENSITIVE 1-associated receptor kinase 1 precursor | - |
| Os04g0140666 | - | Down | 0.70 | Encoding protein kinase | - |
| Os06g0325500 | - | Down | 0.67 | Encoding pantothenate kinase 4 | - |
| Os07g0132500 | - | Down | -0.72 | - | - |
| Os09g0334800 | - | Down | 0.70 | Encoding protein kinase | - |
| Os08g0374600 | *OsRLCK253* | Up | 0.79 | The gene encodes a receptor-like cytoplasmic kinase, which is involved in the regulation of plant abiotic stress resistance. | Giri et al., 2011 |
| Os11g0636900 | *OsU2AF65A* | Up | 0.68 | Encoding splicing cofactor. The mutation of this gene leads to a decrease in the tolerance of rice to high-temperature stress. | Lu et al., 2021 |
| **SA6-2** | | | | | |
| Os05g0588200 | *OsPTD1* | Down | 0.67 | Partner of OsSHOC1. The mutation of this gene results in infertility in both male and female gametophytes, and it exhibits a significant reduction in the number of chromosome exchanges during meiosis. | Ren et al., 2019 |
| Os01g0907400 | *JMJ705* | Down | 0.70 | Encoding H3K27me3 demethylase. This gene is recruited by WOX11 to demethylate H3K27me3 on downstream genes, thereby upregulating the expression of genes involved in the maintenance of apical meristem activity, chloroplast development, and energy metabolism, which promotes normal development of the shoot apex. | Cheng et al., 2018 |
| Os02g0550700 | - | Up | 0.75 | Encoding exosome complex exonuclease | - |
| Os09g0286400 | *OsNHX5* | Up | 0.66 | Encoding vacuolar Na+/H+ antiporter. The gene plays a crucial role in the process of compartmentalizing Na+ and K+ from the cytoplasm to the vacuolar compartment. | Fukuda et al., 2011 |
| Os09g0286300 | - | Down | 0.66 | - | - |
| Os10g0324900 | *OsLHP1* | Up | 0.76 | Heterochronic gene. The mutation of this gene leads to the phenotype of rice with multiple tillers and dwarfed stature, characterized by premature leaf senescence, extended vegetative growth period, and delayed panicle emergence. | Cui et al., 2020 |
| Os03g0783000 | - | Down | 0.68 | Encoding actin | - |
| Os06g0498900 | - | Down | 0.73 | Encoding GTP binding protein | - |
| Os07g0464600 | - | Down | 0.70 | Encoding ATP binding protein | - |
| Os06g0547400 | - | Down | 0.66 | Encoding peroxidase precursor | - |
| Os12g0225200 | - | Up | 0.77 | Encoding outer envelope protein | - |
| Os04g0611400 | - | Up | 0.67 | Encoding vacuolar-sorting receptor precursor | - |
| Os10g0371100 | - | Down | 0.71 | Encoding ethylene-responsive transcription factor | - |
| Os02g0201500 | - | Down | 0.66 | Encoding calcium-binding mitochondrial carrier CBG00135 | - |
| Os04g0394100 | - | Up | 0.65 | Encoding glycerophosphoryl diester phosphodiesterase family protein | - |
| Os11g0129000 | - | Up | 0.67 | Encoding MATE efflux family protein | - |
| Os04g0674600 | - | Up | -0.74 | Encoding transposon protein | - |
| Os11g0172150 | - | Down | 0.69 | Encoding receptor-like protein kinase precursor | - |
| Os04g0393900 | - | Down | 0.65 | Encoding serine/threonine-protein kinase NAK | - |
| Os03g0685300 | *FLO19* | Up | 0.78 | Encoding class I glutamine amidotransferase. The mutation of this gene results in the endosperm of rice appearing white and disrupts the carbon and nitrogen metabolism of rice, affecting its normal growth and development, leading to a reduction in plant height and grain filling rate. | Lou et al., 2021 |
| Os07g0195400 | - | Up | 0.80 | Encoding phosphoacetylglucosamine mutase | - |
| **SC6-6** | | | | | |
| Os04g0321600 | - | Up | -0.74 | Encoding FACT complex subunit SPT16 | - |
| Os03g0620800 | - | Down | 0.67 | Encoding Sec1 family transport protein | - |
| Os08g0130400 | - | Up | -0.71 | Encoding acyl-activating enzyme 14 |  |
| Os04g0321500 | *S28*, *mtRPL27* | Down | -0.74 | Encoding mitochondrial ribosomal protein L27. The mutation of this gene leads to a deficiency in mitochondrial protein synthesis, resulting in respiratory activity defects and the production of sterile pollen. | Yamagata et al., 2010 |

1. Genes corresponding to significantly enriched (P < 0.05) GO terms in the GO enrichment analysis.
2. MITEs located in regulatory regions with methylation levels significantly and strongly correlated (P < 0.05, R > 0.65) to the transgenerational variation in space mutagenic phenotypes.

Cheng, S., Tan, F., Lu, Y., Liu, X., Li, T., Yuan, W., Zhao, Y., Zhou, D. X. (2018). WOX11 recruits a histone H3K27me3 demethylase to promote gene expression during shoot development in rice. Nucleic Acids Res. 46, 2356-2369. doi: 10.1093/nar/gky017

Cui, Y., Cheng, J., Ruan, S., Qi, P., Liu, W., Bian, H., Ye, L., Zhang, Y., Hu, J., Dong, G., Guo, L., Zhang, Y., Qian, Q., Hu, X. (2020). The heterochronic gene Oryza sativa LIKE HETEROCHROMATIN PROTEIN 1 modulates miR156b/c/i/e levels. J Integr Plant Biol. 62, 1839-1852. doi: 10.1111/jipb.12991

Fukuda, A., Nakamura, A., Hara, N., Toki, S., Tanaka, Y. (2011). Molecular and functional analyses of rice NHX-type Na+/H+ antiporter genes. Planta. 233, 175-188. doi: 10.1007/s00425-010-1289-4

Giri, J., Vij, S., Dansana, P. K., Tyagi, A. K. (2011). Rice A20/AN1 zinc-finger containing stress-associated proteins (SAP1/11) and a receptor-like cytoplasmic kinase (OsRLCK253) interact via A20 zinc-finger and confer abiotic stress tolerance in transgenic Arabidopsis plants. New Phytol. 191, 721-732. doi: 10.1111/j.1469-8137.2011.03740

Konishi, N., and Ma, J. F. (2021). Three polarly localized ammonium transporter 1 members are cooperatively responsible for ammonium uptake in rice under low ammonium condition. New Phytol. 232, 1778-1792. doi: 10.1111/nph.17679

Li, K., Zhang, S., Tang, S., Zhang, J., Dong, H., Yang, S., Qu, H., Xuan, W., Gu, M., Xu, G. (2022). The rice transcription factor Nhd1 regulates root growth and nitrogen uptake by activating nitrogen transporters. Plant Physiol. 189, 1608-1624. doi: 10.1093/plphys/kiac178

Lou, G., Chen, P., Zhou, H., Li, P., Xiong, J., Wan, S., Zheng, Y., Alam, M., Liu, R., Zhou, Y., Yang, H., Tian, Y., Bai, J., Rao, W., Tan, X., Gao, H., Li, Y., Gao, G., Zhang, Q., Li, X., Liu, C., He, Y. (2021). FLOURY ENDOSPERM19 encoding a class I glutamine amidotransferase affects grain quality in rice. 41: 36. doi: 10.1007/s11032-021-01226-z

Lu, S., Gao, C., Wang, Y., He, Y., Du, J., Chen, M., Zhao, H., Fang, H., Wang, B., Cao, Y. (2021). Phylogenetic Analysis of the Plant U2 snRNP Auxiliary Factor Large Subunit A Gene Family in Response to Developmental Cues and Environmental Stimuli. Front Plant Sci. 12:739671. doi: 10.3389/fpls.2021.739671

Nakamura, A., Fujioka, S., Sunohara, H., Kamiya, N., Hong, Z., Inukai, Y., Miura, K., Takatsuto, S., Yoshida, S., Ueguchi-Tanaka, M., Hasegawa, Y., Kitano, H., Matsuoka, M. (2006). The role of OsBRI1 and its homologous genes, OsBRL1 and OsBRL3, in rice. Plant Physiol. 140, 580-590. doi: 10.1104/pp.105.072330

Niu, M., Wang, H., Yin, W., Meng, W., Xiao, Y., Liu, D., Zhang, X., Dong, N., Liu, J., Yang, Y., Zhang, F., Chu, C., Tong, H. (2022). Rice DWARF AND LOW-TILLERING and the homeodomain protein OSH15 interact to regulate internode elongation via orchestrating brassinosteroid signaling and metabolism. Plant Cell. 34, 3754-3772. doi: 10.1093/plcell/koac196

Park, C. J., Wei, T., Sharma, R., Ronald, P. C. (2017). Overexpression of Rice Auxilin-Like Protein, XB21, Induces Necrotic Lesions, up-Regulates Endocytosis-Related Genes, and Confers Enhanced Resistance to Xanthomonas oryzae pv. oryzae. Rice. 10, 27. doi: 10.1186/s12284-017-0166-1

Ren, Y., Chen, D., Li, W., Zhou, D., Luo, T., Yuan, G., Zeng, J., Cao, Y., He, Z., Zou, T., Deng, Q., Wang, S., Zheng, A., Zhu, J., Liang, Y., Liu, H., Wang, L., Li, P., Li, S. OsSHOC1 and OsPTD1 are essential for crossover formation during rice meiosis. (2019). Plant J. 98, 315-328. doi: 10.1111/tpj.14214

Sonoda, Y., Ikeda, A., Saiki, S., Yamaya, T., Yamaguchi, J. (2003). Feedback regulation of the ammonium transporter gene family AMT1 by glutamine in rice. Plant Cell Physiol. 44, 1396-1402. doi: 10.1093/pcp/pcg169

Yamagata, Y., Yamamoto, E., Aya, K., Win, K. T., Doi, K., Sobrizal., Ito, T., Kanamori, H., Wu, J., Matsumoto, T., Matsuoka, M., Ashikari, M., Yoshimura, A. (2010). Mitochondrial gene in the nuclear genome induces reproductive barrier in rice. Proc Natl Acad Sci USA. 107, 1494-1499. doi: 10.1073/pnas.0908283107

Yamamuro, C., Ihara, Y., Wu, X., Noguchi, T., Fujioka, S., Takatsuto, S., Ashikari, M., Kitano, H., Matsuoka, M. (2000). Loss of function of a rice brassinosteroid insensitive1 homolog prevents internode elongation and bending of the lamina joint. Plant Cell. 12, 1591-1606. doi: 10.1105/tpc.12.9.1591

Zhao, J., Wu, C., Yuan, S., Yin, L., Sun, W., Zhao, Q., Zhao, B., Li, X. (2013). Kinase activity of OsBRI1 is essential for brassinosteroids to regulate rice growth and development. Plant Sci. 199-200, 113-120. doi: 10.1016/j.plantsci.2012.10.011
