# Supplementary material for: Miniature-inverted-repeat transposable elements contribute to phenotypic variation regulation of rice induced by space environment
Source: Front Plant Sci. 2025 Jan 8;15:1446383. doi: 10.3389/fpls.2024.1446383 (PMC11751223; doi:10.3389/fpls.2024.1446383)
Supplement: Supplementary Figure 1 — Breeding process of rice space-mutagenic lines using a pedigree method. [file DataSheet1.zip › Supplementary Material/Supplementary Table 9.docx]

Supplementary Table 9. Genes associated with phenotypic regulation in the vicinity of space induced MITEs-TIPs

| **Space mutagenic lines** | **GO Terms** | **MITE-TIPs Location** | **Gene ID** | **Gene Symbols** | **Function** | **Reference** |
| --- | --- | --- | --- | --- | --- | --- |
| **SA3-7** | plant epidermis development、  shoot system development | Down | Os07g0132300 | *OsEPFL5* | *OsEPF/EPFL* family genes encode small secretory signal peptides that play regulatory roles in plant growth and development. Some of these *OsEPF/EPFL* genes play important roles in awn development. | Xiong et al., 2022; Guo et al., 2023 |
| **SA6-2**  **SC6-6** | shoot system development | Up | Os09g0395300 | *SLL1*; *AH2* | *SLL1* encodes MYB domain protein, and its dysfunction may lead to enhanced leaf rolling and photosynthesis in rice, as well as smaller grain size and altered grain quality, and may play a role in multiple aspects of plant development. | Zhang et al., 2009; Ren et al., 2019 |
|  | regulation of growth、regulation of immune response | Down | Os05g0333200 | *D1*; *RGA1*; *D89* | Recessive mutations in *D1* may cause dwarf rice, which is also accompanied by other traits. This gene is involved in drought tolerance in the vegetative stage of rice, and its mutant are less sensitive to drought stress. | Ferrero-Serrano and Assmann., 2016; Ferrero-Serrano and Assmann., 2018; Li et al., 2023 |
